# False Positive 18F-FDG Positron Emission Tomography Findings in Schwannoma—A Caution for Reporting Physicians

**DOI:** 10.3389/fmed.2018.00275

**Published:** 2018-10-08

**Authors:** Paul Boré, Renaud Descourt, Luc Ollivier, Pierre-Yves Le Roux, Ronan Abgral

**Affiliations:** ^1^Service d'Oncologie Médicale, Centre Hospitalier Régional et Universitaire de Brest, Université de Bretagne Occidentale, Brest, France; ^2^Service de Radiothérapie, Centre Hospitalier Régional et Universitaire de Brest, Université de Bretagne Occidentale, Brest, France; ^3^Service de Médecine Nucléaire, EA 3878 (GETBO) IF 148, Centre Hospitalier Régional et Universitaire de Brest, Université de Bretagne Occidentale, Brest, France

**Keywords:** positron-emission tomography, schwannoma, neoplasm staging, ovarian neoplasms, mediastinal neoplasms

## Abstract

Schwannoma is a rare source of false-positive 18F-fluorodeoxyglucose (18F-FDG) uptake in Positron-emission tomography (PET/CT), inducing potential errors in staging of several solid cancer, with implications for patient management. This clinical case reports the situation of a patient undergoing an 18F-FDG-PET/CT for initial staging of an ovarian adenocarcinoma. We found a high paramediastinal hypermetabolic mass suspicious of remote extension or secondary synchronous primitive tumor. The biopsy finally reveals a histopathology of Schwannoma, allowing the patient to be eligible for a surgical procedure of her ovarian adenocarcinoma by rejecting the hypothesis of malignancy.

## Background

Even if they are considered as benign tumors, high FDG uptake can be seen in schwannomas, providing a risk of false-positive interpretation with consequences on tumor staging and also management of patient.

For example Martinez-Esteve et al. (1) have reported the case of a patient with a locally advanced HER2 overexpressed breast tumor treated by chemotherapy and Trastuzumab showing a hypermetabolic retro-tracheal lymphadenopathy [15 × 17 mm and standardized uptake value (SUVmax) 4.5]. On the 18F-FDG PET-CT intermediate therapeutic assessment, there was a complete metabolic response except for the retro tracheal lymphadenopathy. The histopathological analysis of this finally excluded a metastatic lesion related to breast cancer and revealed a benign Schwannoma, allowing to continue initial therapeutic strategy.

Moreover, Gorospe et al. (2) have published a case of difficult initial staging of a pulmonary adenocarcinoma of the right upper lobe. Indeed, 18F-FDG PET/CT findings concluded to a suspicious supraclavicular right lymph node (SUVmax 2.6 vs. 2.8 for the primary) and the patient's tumor was restaged into a T3N3M0 IIIB lung cancer vs. T3N0M0 IIB according to WHO classification (3). Before modifying treatment management of patient based on 18F-FDG PET/CT findings, a surgical biopsy was performed and finally concluded again to a schwannoma.

Several other case reports show that schwannomas are usually associated with a high 18F-FDG uptake on PET-CT (4–6). But, cases of schwannomas with low uptake exist even if they are less frequent (7).

Only few cases of schwannoma located in the mediastinum with an 18F-FDG uptake have already been reported in literature.

## Case presentation

A 75-year-old female patient with previous hystory of active smoking at 75 year-package associated with other cardiovascular risk factors (hypertension, hypercholesterolemia, non-insulin-dependant diabetes, and obesity) has been sent to our university hospital for a suspicion of strangulated umbilical hernia. An abdomen and pelvis CT scan was then performed and found a diffuse infiltration of mesenteric fat evoking a peritoneal carcinosis without primary tumor clearly identified.

An exploring laparoscopy showed a visual aspect of inflammatory peritoneum with a thickened epiploon and non-tumorous ovaries. On the contrary, histopathological examinations (biopsy and cytology) suggested an immunohistochemical profile compatible with high-grade serous papillary carcinoma of ovarian or peritoneal origin. The therapeutic strategy included neo-adjuvant chemotherapy by CARBOPLATIN-PACLITAXEL and interval surgery after 3 cycles.

**Figure 1:**
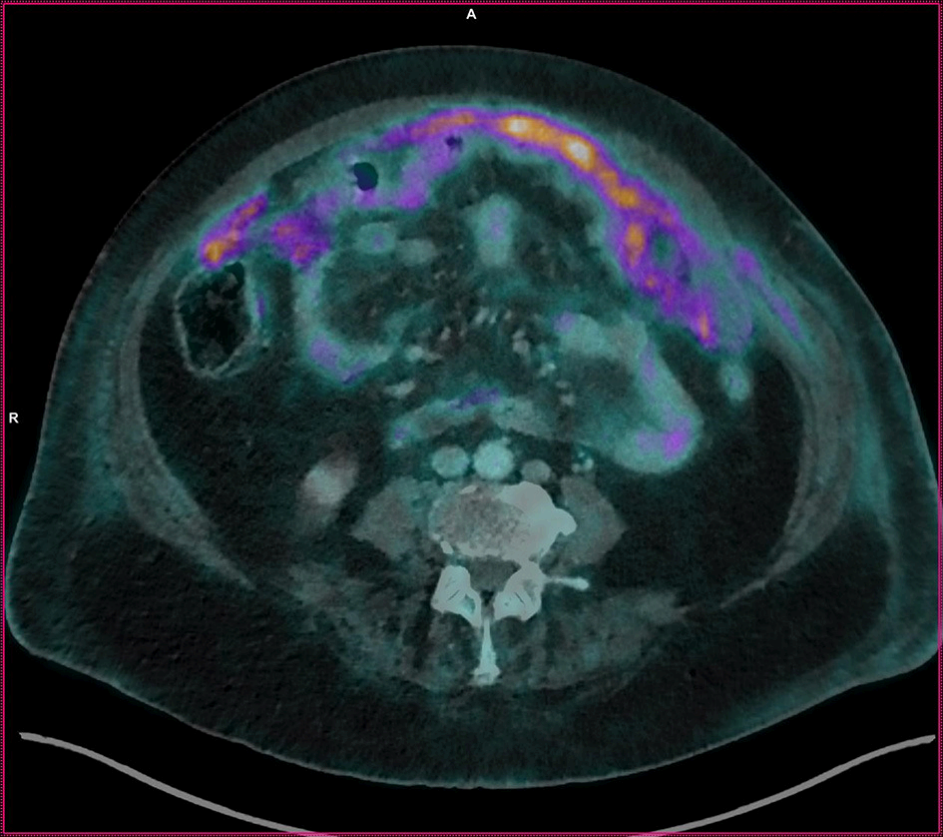
Fusion image in cross section. Image of peritoneal carcinomatosis of the patient.

**Figure 2:**
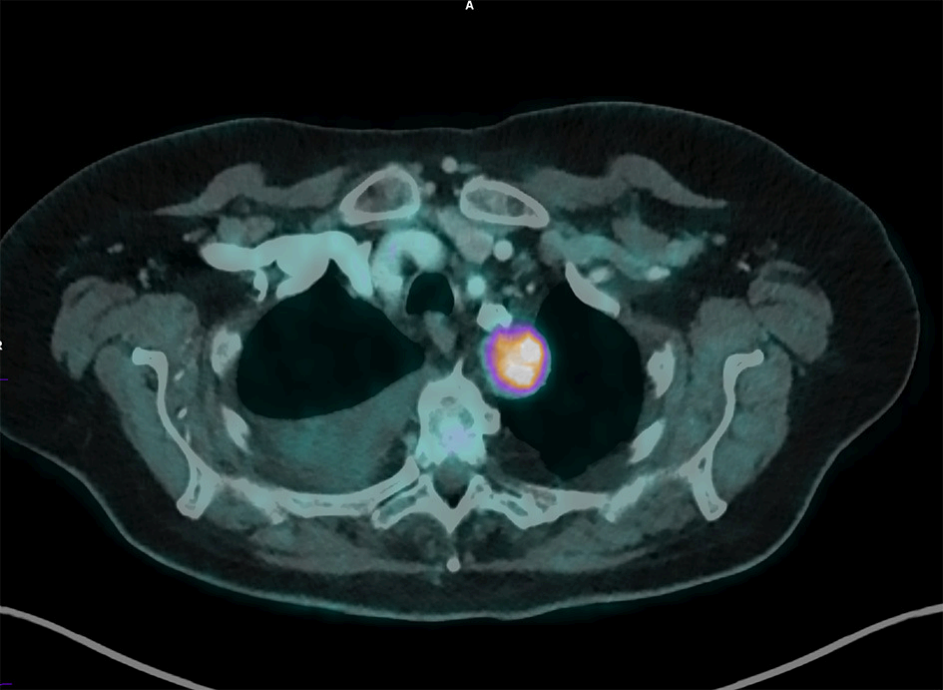
Fusion image in axial cut. It is found that the mass is well-located behind the tracheabronchial axis.

**Figure 3:**
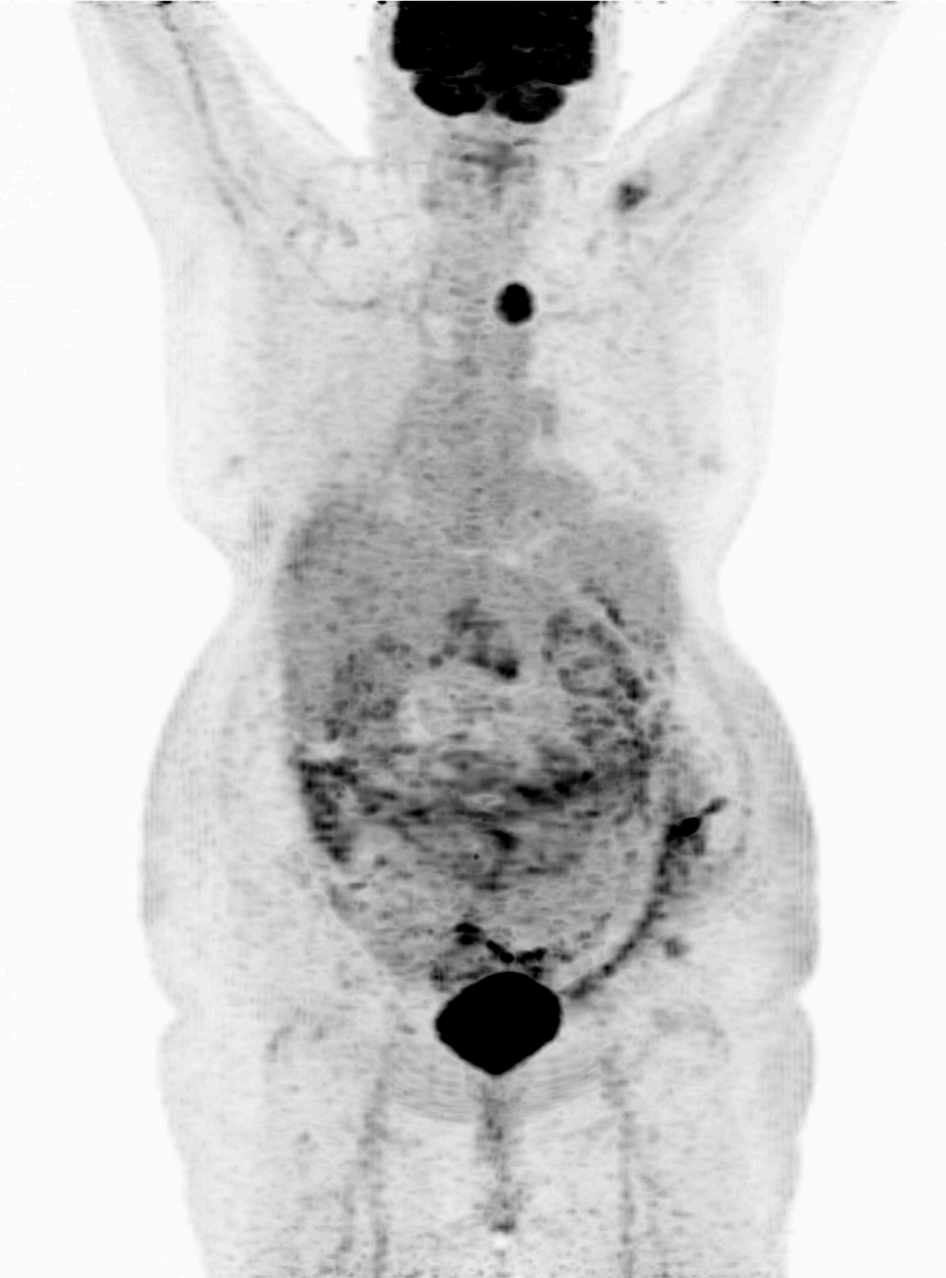
Maximal intensity projection of our patient. Acquisition of broadcoast images realized 60 min after injection of 252 Megabecquerels of FDG-IBA in a vein of the right wrist. Visualization of the supra-diaphragmatic isolated hypermetabolism.

**Figure 4:**
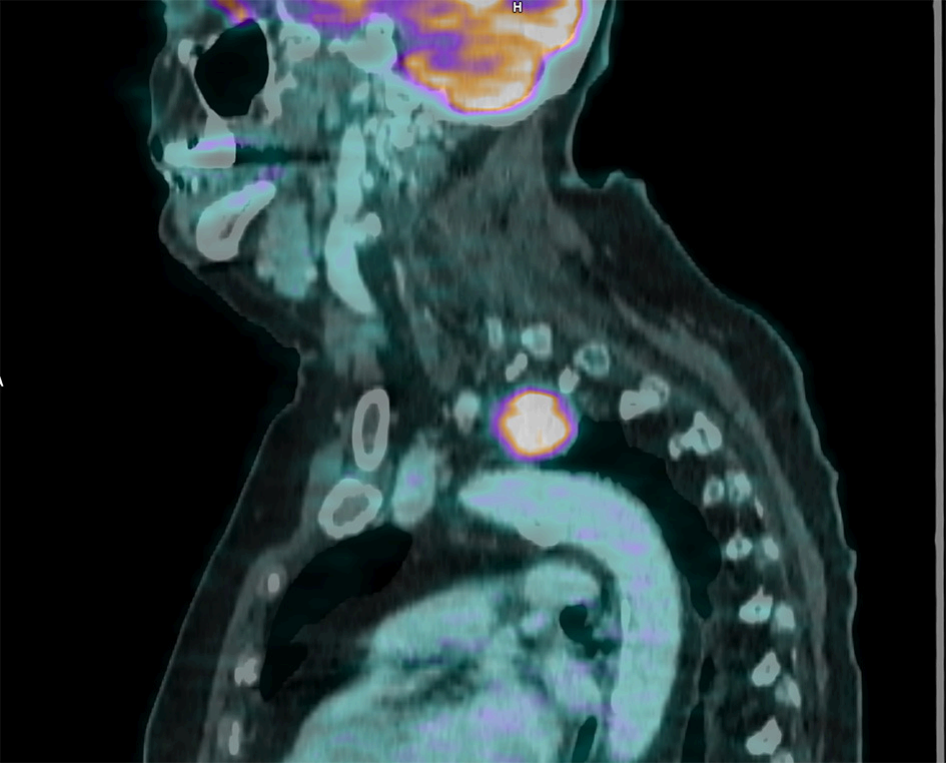
Sagittal cut, fusion image, 30 mm mass above aortic stock, maximum standardized uptake value of 12.8.

Moreover, an 18F-fluorodeoxyglucose (18F–FDG) Positron-emission tomography (PET/CT) was performed not to ignore a supra-diaphragmatic remote extension of disease that would exclude surgery indication. In addition to multiple hypermetabolic known peritoneal carcinomatosis lesions (Figure 1), PET CT found fortuitly a pathological 18F-FDG uptake upon a high paramediastinal tissue 3 cm mass located at the left pulmonary apex (SUV max: 12.8) (Figures 2–4). Due to this suspicion of remote extension of disease or secondary primary tumor, a biopsy under CT scan was performed. The histolopatological analysis concluded with an appearance of Schwannoma, without any sign suggestive of malignancy.

While awaiting the histological characterization of this mass, the patient finally benefited from 6 cycles of chemotherapy before surgery by laparotomy. Unfortunately due to carcinomatosis extended to the entire abdominal cavity with a peritoneal index at 19 (8) a complete resection surgery was not possible and new courses of CARBOPLATIN TAXOL were scheduled.

## Discussion

Schwannomas are the most common nerve sheath tumors (9) and are usually solitary. Majority of lesions are benign tumors that usually grow slowly [only 2% of them may evolve into a malignant form (10, 11)]. They are often asymptomatic and fortuitly revealed. Microscopically they are well circumscribed with a surrounding capsule and are composed by a clonal population of Schwann cells. There are two components, a highly ordered cellular called Antoni A area (hypercellular area) and a looser myxoid component called Antoni B area. In immunohistochemistry they express the S100 protein (12). Most of them are sporadic tumors. However in few cases, they are associated with neurofibromatosis type 2 disease, Carney's complex, or schwannomatosis. There are also some variants of schwannomas which are cellular schwannoma, plexiform schwannoma, and melanotic schwannoma (13).

This tumor consists of an abnormal proliferation of Schwann cells developped from nerves. The most frequent locations of schwannomas are the brachial plexus or the large nerve trunks of the limbs (particularly elbow, wrist, or knee). Deep forms in retroperitoneum or mediastinum exist and are often of large size (14). In the mediastinum, nerve tumors such as schwannoma are located in the posterior compartment where they account for two-third to 80% of tumors in this localization (15–17). The other most common masses of the posterior mediastinum are meningoceles, para esophageal cysts, goiter, and lymphoma. Three compartments are described in the mediastinum. The posterior mediastinum is the paravertebral zone bounded by the posterior trachea and pericardium, the diaphragm, the vertebral column, and the thoracic inlet (18). The anterior mediastinum is the prevascular zone and the middle mediastinum is the peri-tracheoesophageal zone (17). Regarding the other compartments, lymphoma, germinal tumor, and thymic tumors are typically located in the anterior (19) and lymphadenopathy and lymphoma in the middle mediastinum.

On imaging, schwannomas occur on the path of a nerve, are well-limited, often oval and unique. The metabolic characteristics of schwannomas on 18F-FDG PET-CT are not well-described in literature to differentiate benign or malignant forms. Ahmed et al. did not found an association between SUV and benign schwannoma or malignant tumor (20).

Beaulieu et al. (21) compared the FDG uptake with Schwannoma cellularity, tumor size and tumor proliferation rate (Ki-67 index). The SUVmax varied from 1.9 to 7.2 (mean = 4.6; *n* = 9). They found that the SUV max of the hypocellular tumors was significantly lower than the SUV max of the hypercellular tumors (composed by a larger; *p* = 0.010). They did not found an association between SUVmax and tumor size or Ki-67 index. They did not find why there is a high FDG accumulation in benign tumors such as schwannoma.

In a study of 22 histologically proven schwannomas, Miyake et al. (22) did not find a correlation between expression of glucose transporters (GLUTs) and SUVmax of the lesions that could explain why there is a high FDG accumulation in this tumors. Moreover, SUVmax ranged from 1.5 to 17.3 with a median of 3.7 and factors significantly associated with higher SUVmax were gastrointestinal origin (*p* = 0.007) and the presence of peri-tumor lymph nodes (*p* < 0.001).

More recently it has been showed that schwannomas with18F-Dihydroxyphenylalanine (DOPA) uptake (23) and also in 68Ga-Prostate-specific membrane antigen (PSMA) PET-CT (24).

It therefore seems difficult to conclude to a diagnosis of Schwannoma only based on SUV characteristics on PET-CT since their SUVmax seems to vary enormously. There is also several other causes of known false positives such as infectious and inflammatory processes.

Several cases reporting masses to PET-CT with hypermetabolism in follows-up of tumoral pathologies have been described, and these hypermetabolisms are generally associated with malignant tumors. However as described earlier that is not always true. Schwannomas with an 18F-FDG uptake have been described but especially outside the mediastinum. To our knowledge only few publications report this situation in the mediastinum. This case confirms the necessity to be aware of a neurogenic tumor when the PET reader concludes to a hypermetabolic mass of the posterior mediastinum and especially if it can lead to a change in patient care.

## Concluding remarks

Schwannoma is a potential cause of 18F-FDG false positive uptake in PET-CT, inducing risk of worse staging or therapeutic assessment, with consequences on patient management. Currently there are no reliable argument to differentiate a benign Schwannoma from a malignant tumor only on 18F-FDG-PET-CT imaging. This case report also recalls the need to biopsy doubtful lesions fortuitly discovered on 18F-FDG-PET-CT in order to avoid a loss of chance and possible complications related to unnecessary treatment toxicities.

## Ethics statement

All procedures performed in this study were in accordance with the ethical standards of the institutional research committee on human experimentation and with the Helsinki Declaration of 1975, as revised in 2008. Ethical review and approval was not required in accordance with the national and institutional requirements. The patient provided written informed consent.

## Author contributions

PB provided details of the patient and provided initial draft of submission. RD provided details of the patient and helped draft the initial submission. P-YL and RA provided images, image analysis, and helped draft the initial submission. LO helped draft the initial submission.

### Conflict of interest statement

The authors declare that the research was conducted in the absence of any commercial or financial relationships that could be construed as a potential conflict of interest.
